# Two Out of Three Ain’t Bad: Aggregating Multiple Identification Decisions from the Same Observer Improves Unfamiliar Face Matching Performance

**DOI:** 10.1177/17470218261452983

**Published:** 2026-05-13

**Authors:** Daniel J. Carragher, Peter J. B. Hancock

**Affiliations:** 1 School of Psychology, College of Education, Behavioural and Social Sciences, Adelaide University, SA, Australia; 2 Psychology, Faculty of Natural Sciences, University of Stirling, Scotland, UK

**Keywords:** identity verification, face matching, identification, combined decision-making, wisdom of crowds

## Abstract

Deciding whether two images show the same person sounds easy, but the average individual achieves accuracy of 60% to 90% on many unfamiliar face matching tasks. However, overall task performance can be improved by combining the identification decisions made by different individuals through the “wisdom of the crowd” effect. We investigated whether similar gains occur when combining multiple decisions made by the same individual, via a “wisdom of the crowd within” effect. Participants completed the same unfamiliar face matching task three times: in one session (Experiment 1), or over 3 weeks (Experiment 2). We found a wisdom of the crowd within effect in both experiments, which became significant after two responses and was larger with three. Surprisingly, the delay did not offer additional benefit. Though the crowd within improved performance, crowds of different individuals produced larger gains. Our results contribute to a growing literature showing that combined decision-making can improve unfamiliar face matching performance.

## Introduction

Identity verification often occurs as a one-to-one face matching task, in which an observer compares an individual’s appearance to that of their photo-ID document (Bruce et al., 1999; Fysh & Bindemann, 2017; Kemp et al., 1997; Megreya & Burton, 2006). Despite the prevalence of this task, average human accuracy on laboratory tests of unfamiliar face matching often ranges from 60% to 90% (Burton et al., 2010; Carragher & Hancock, 2020; Dowsett & Burton, 2015; Fysh & Bindemann, 2018; Stantic et al., 2022; White et al., 2022). This level of average accuracy occurs even though both images are presented simultaneously, often without enforced time restrictions, and the images themselves are generally high quality. There are, however, significant differences in face matching ability, with some individuals demonstrating exceptionally high performance, while others perform near chance (Bobak et al., 2016; Burton et al., 2010; Estudillo et al., 2021; Robertson et al., 2016). Some highly trained professionals outperform novices under certain conditions (Phillips et al., 2018; White et al., 2015), but professional experience does not guarantee exceptional face matching ability (White et al., 2014; Wirth & Carbon, 2017). Training courses lasting hours or days may lead to modest improvements (around 6%) to an individual’s face matching performance (Carragher et al., 2022; Towler et al., 2021b), or to no improvement at all (for review, see Towler et al., 2021a).

Although training individuals to become better at face matching has had limited success (Towler et al., 2019, 2021a), completing an unfamiliar face matching task in a pair with another person can improve performance (Bruce et al., 2001; Dowsett & Burton, 2015; Ritchie et al., 2022). Interestingly, gains of a similar magnitude are realised even when these pairs are formed statistically and do not have social interaction (Jeckeln et al., 2018). Such pairs do not even have to be human, with gains also achieved by statistically fusing independent decisions made by different facial recognition algorithms (Towler et al., 2023), as well as those of humans and facial recognition algorithms (Kramer, 2025; O’Toole et al., 2007; Phillips et al., 2018); though decisions made by humans interacting with highly accurate algorithms are often suboptimal (Bartlett et al., 2024; Carragher & Hancock, 2023; Carragher et al., 2024; Hua et al., 2025). The benefits of this combined decision-making approach to unfamiliar face matching are consistent with the “wisdom of the crowd” effect (Galton, 1907), in which averaging independent decisions or judgements from many individuals leads to an overall crowd response that is more accurate than the vast majority of individual responses.

The wisdom of the crowd effect has been observed in a wide variety of domains (see Surowiecki, 2005), including in perceptual forensic judgements such as fingerprint analysis (Tangen et al., 2020), post-mortem identification (Davis et al., 2019), and distinguishing real from computer generated faces (Kramer & Cartledge, 2024). Importantly, the wisdom of the crowd also improves unfamiliar face matching performance. White et al. (2013) first showed that combining the identification decisions made independently by multiple individuals led to performance gains of around 20% in an unfamiliar face matching task, when compared to the average individual performance of the same participants on the same task. In a face matching task with binary responses (i.e., “same/different”), the wisdom of the crowd can emerge using a simple “majority rules” decision rule,^1^ so long as the majority of crowd members respond correctly to a given trial (e.g., 12 correct responses and 8 incorrect responses is enough for a crowd of 20 to answer a trial correctly, even though 40% of individuals were incorrect). Thus, a crowd can achieve high levels of accuracy over the course of an entire face matching task, even with completely average levels of individual performance – provided the crowd members make different mistakes from each other. Carragher and Hancock (2024) have extended this work to show that the wisdom of the crowd effect can improve face matching performance by 11% to 26% on a challenging task with faces shown wearing surgical masks. The wisdom of the crowd is an effective approach to improve overall performance on unfamiliar face matching tasks (Balsdon et al., 2018; Carragher & Hancock, 2024; Cavazos et al., 2023; Davis et al., 2019; Jeckeln et al., 2018; Kramer & Javorková, 2025; White et al., 2015).

Although the wisdom of the crowd effect is commonly attributed to the independence of the decisions made by different observers, research has shown that the responses do not have to come from different individuals. Vul and Pashler (2008) asked their participants to complete a general knowledge quiz that required numeric answers. On completion, half of the participants were asked to complete the same quiz immediately, while the other half completed the quiz again 3 weeks later. The authors found that averaging the two answers provided by the same participants to the same questions led to reduced error compared to either individual answer, much like Galton’s (1907) original study. Interestingly, the authors reported that the benefit of averaging these decisions was greater when the second response was made after a 3-week delay, which was attributed to increasing independence between the responses (i.e., participants were less likely to remember their previous response). Vul and Pashler’s (2008) results demonstrated that it is possible to observe wisdom of the crowd effects in repeated responses by a single individual – the “wisdom of the crowd within.” While the wisdom of the crowd within has since been demonstrated in many different domains (for review, see Herzog & Hertwig, 2014), at the time we pre-registered the current project, no one had tested whether the effect extended to unfamiliar face matching performance.

Wisdom of the crowd effects require variability in the responses of the crowd members. There would be no benefit to combining responses that were identical. As described, individuals differ in their face matching abilities (Bobak et al., 2016; Burton et al., 2010). As such, a minority of participants will make an error on an “average” face matching trial, while the majority will give the correct response, providing the variability needed for the conventional wisdom of the crowd effect to occur (Carragher & Hancock, 2024; Kramer & Javorková, 2025; White et al., 2013). However, less is known about the variability of face matching performance within an individual, because participants rarely complete the same test more than once. But by examining the test-retest reliabilities reported with some standardised face matching tests, we see that although average task performance across all participants is generally highly similar at Test 1 and Test 2, the correlation of individual performance in the two sessions is not perfect. The short Kent Face Matching Task (KFMT) reported a correlation of *r* = .67 for test-retest performance (Fysh & Bindemann, 2018), while correlations of *r* = .77 (short test) and *r* = .78 (long test) were reported for the Glasgow Face Matching Test 2 (GFMT2; White et al., 2022), and Kramer et al. (2021) reported a test-retest correlation of *r_s_* = .65 for the short version of the original Glasgow Face Matching Test (GFMT: Burton et al., 2010). Though high, these correlations in test-retest performance demonstrate a point that is often overlooked – the same observer must occasionally change the identification decision they make for the same pair of faces.

In one of the few studies to examine the consistency of an individual’s face matching responses, Bindemann et al. (2012: Experiment 1) asked participants to complete the same 200-trial face matching task on three consecutive days. The authors found that average accuracy was approximately 90% on all 3 days. Yet, the number of trials resolved correctly on consecutive days declined as the experiment progressed; from approximately 90% on day 1, to 80% to 85% answered correctly on both day 1 and day 2, and 75% to 80% answered correctly all 3 days. Thus, even though participants achieved the same level of average performance each day, they did so by answering different trials correctly each time. Crucially, Bindemann et al. (2012) found that participants were much more likely to make a single error for a given face pair than they were to make the same mistake two or three times. If one were to combine these three responses with the simple “majority rules” decision threshold described above, the trials that were answered correctly two out of three times would be resolved correctly, which would lead to a wisdom of the crowd within effect in these data.

Our original aim for the current project was to investigate whether the wisdom of the crowd within could improve performance in an unfamiliar face matching task. While such an effect was hinted at in previous data (Bindemann et al., 2012), it had not been formally reported or tested, making this a novel investigation at the time we submitted our pre-registrations and began data collection. Shortly after we started our project, Kramer et al. (2023) published a study showing that the wisdom of the crowd within does indeed improve unfamiliar face matching performance. Their participants completed the short GFMT (Burton et al., 2010) twice in the same testing session. The authors also re-analysed previous data (Kramer et al., 2021), in which participants had completed the short GFMT twice, but at least 12 days apart. Rather than making binary identification responses (i.e., same/different), the participants made responses on a 5-point scale, which allowed the authors to test for crowd effects among crowds of 2 (see footnote 1). Kramer et al. (2023) found that averaging the two responses made by the same participant to each trial led to improved overall performance – a wisdom of the crowd within effect – in both experiments.^2^

Although Kramer et al.’s (2023) timely paper answered our initial pre-registered research question by demonstrating the wisdom of the crowd within for unfamiliar face matching performance, our current project still makes several important contributions to this line of research. First, our participants completed the same face matching task three times, meaning we can test whether the wisdom of the crowd within effect increases beyond two responses. Second, by recruiting larger samples of participants, we have greater statistical power to re-examine two of Kramer et al.’s (2023) findings: whether the conventional wisdom of the crowd effect (different individuals) is larger than the wisdom of the crowd within, and whether the wisdom of the crowd within increases with a greater delay between repeated responses. Both effects were reported by Vul and Pashler (2008) in their study that used general knowledge questions as stimuli, but were not found by Kramer et al. (2023) for unfamiliar face matching (or not tested directly for concerns of low statistical power). Third, we used a different face matching task and a different response scale than Kramer et al. (2023), which is important for demonstrating the generalisability of the wisdom of the crowd within effect. In all, our project complements and extends Kramer et al.’s (2023) investigation of the wisdom of the crowd within for unfamiliar face matching.

### Pre-Registrations and Data

The design, hypotheses, and analysis plan for our two experiments were pre-registered prior to data collection on the Open Science Framework (OSF): Experiment 1 (https://osf.io/tbgy3/overview) and Experiment 2 (https://osf.io/shw5u/overview). All data analysed in this project are available in the same repository, as are our Supplemental Material (https://osf.io/wsv5f/overview).

## Experiment 1

We start by investigating whether the wisdom of the crowd within effect emerges when participants complete the same unfamiliar face matching task three times in a single testing session. We predicted **(H1)** that aggregating multiple responses from the same individual to the same stimuli would lead to greater performance than their individual performance in a single test, which would constitute a wisdom of the crowd within effect (Bindemann et al., 2012). Consistent with previous research (White et al., 2013), we expected **(H2)** that aggregating responses from different individuals would result in a conventional wisdom of the crowd effect. Finally, based on Vul and Pashler (2008), we predicted **(H3)** that the wisdom of the crowd effect would be larger for crowds of different individuals (“between crowds”) than for crowds of the same individual (“within crowds”).

### Method

#### Sample Size

To guide our choice of sample size during the pre-registration process, we examined open access data published by Alenezi et al. (2015) who, in studying unfamiliar face matching performance over time, asked participants (*N* = 50, combining Experiments 1 and 2) to complete the same task five times in a single session. Our novel analysis of these data revealed a wisdom of the crowd within effect (the majority decision of their five responses was more accurate than their first response in Block 1), with a large effect size for the signal detection measure of sensitivity (*d*′; *p* < .001, Cohen’s *d* = 1.125), but a smaller one for overall accuracy (*p* = .054, *d* = 0.279). Although our primary measure in the current study will be area under the curve (AUC), which is a measure of sensitivity like *d*′, we based our power analysis on the effect size returned for overall accuracy, to ensure that we would have appropriate power to detect smaller effects.

An a priori power analysis using G*Power 3.1 software (Faul et al., 2007, 2009) revealed that 104 participants would be required to achieve 80% power to detect an effect size of Cohen’s *d* = 0.279 in a paired samples *t*-test with a conventional alpha of .05. Therefore, we aimed to recruit 120 participants, expecting to have data from approximately 100 participants once our pre-registered exclusion criteria had been applied.

#### Participants

We received consent from 125 unique participants, who were recruited from *Prolific* (www.prolific.com), a research participation website commonly used for experimental psychology studies (Palan & Schitter, 2018). Participants were eligible if they lived in the United States and reported fluency in English. Following our pre-registered exclusion criteria, we removed data from participants who did not complete the task (*n* = 6), attempted the experiment more than once (*n* = 2), failed an attention check trial (*n* = 1), or took longer than 60 min to complete the task (*n* = 3). Our final sample consisted of 113 participants (*M*_age_ = 37.8, *SD* = 13.7; 53 female, 56 male, 3 non-binary, and 1 non-disclosed gender identity). The entire experiment took an average of 22.7 min to complete (*SD* = 8.7), and all participants received 3.10 GBP (approximately 3.76 USD) for their time. This project received ethical approval (approval no. #22/57) from the Human Research Ethics Subcommittee in the School of Psychology at the University of Adelaide.

#### Procedure

Participants were told that they would be completing a face matching task, wherein they would be asked to decide whether two simultaneously presented faces showed the same person or two different people. The participants completed the short version of the GFMT2, which consists of 80 trials in total, of which half are identity matches (White et al., 2022). Participants responded using a 6-point scale, which conveyed their identification decision (“same” or “different”) and confidence (“definitely,” “probably,” “guess”). Responses were coded from 1 “definitely different” to 6 “definitely same.” Each face pair remained onscreen until a response was made, without restriction.

All participants completed the GFMT2-S three times in a single experimental session. The experiment was divided into three blocks, each of which contained the entire GFMT2-S task. Participants completed a full block before starting the next block. Trial order was randomised within each block. Participants could take a short, self-paced break between blocks.

#### Measures of Performance

##### Area Under the Curve

Our primary measure of performance is AUC (calculated using *perfcurve* in MATLAB 24.2.0, The MathWorks Inc.), a measure of sensitivity from signal detection theory that shows how well participants can distinguish identity match trials from mismatch trials at differing response thresholds (Macmillan & Creelman, 2004; Stanislaw & Todorov, 1999). An AUC of 1.0 indicates perfect discrimination performance, whereas 0.5 signals chance performance. We pre-registered AUC as our primary measure of performance because it makes full use of our 6-point response scale.

##### Overall Accuracy

We also report overall accuracy as a second measure of performance to ensure our results are comparable to previous research. To calculate overall accuracy, the identification decisions made using the 6-point response scale were converted to numerical values, in which 1 to 3 represent “different” identity judgements (definitely, probably, and guess), and 4 to 6 represent “same” identity responses (guess, probably, and definitely). Values to the true side of 3.50 were considered a “correct” response to a given trial (mismatch trials <3.50, match trials >3.50). Values of exactly 3.50, which can occur when averaging decisions together, were resolved by a random coin-flip procedure (see Carragher & Hancock, 2024). In Experiment 1, resolution was required for 8.99% of trials in Block 2 and none in Block 3 (it is not possible to obtain 3.5 by averaging three responses of 1–6).

##### Criterion

Finally, we report an analysis of criterion to provide additional context around the interpretation of overall accuracy (AUC is a measure of performance independent of criterion). Criterion is a measure of response bias (Macmillan & Creelman, 2004; Stanislaw & Todorov, 1999), wherein negative values represent a liberal threshold that results in a bias towards responding “same” (which will increase accuracy on match trials), and positive values indicate a conservative threshold that corresponds to making more “different” decisions (increasing accuracy for identity mismatches). A value of zero indicates no bias.

##### Supplemental Material

We report a summary of *d*′, another measure of sensitivity from signal detection theory, in our Supplemental Material as an additional measure of performance. We also include analysis of the optimal AUC threshold (for separating match and mismatch trials), which is a measure returned by the AUC function that can contribute to the interpretation of response bias. Our Supplemental Material is available in our OSF repository.

#### Creating Crowds

##### Within Crowds

We created cumulative crowds for Block 2 and Block 3 by averaging the response an individual gave most recently with all those they had made previously for the same trial. The average response value (1–6) was taken as the decision of the “within crowd” to a single trial. We then calculated the same measures of performance (AUC, overall accuracy) for each within crowd as we did for each individual participant. Please note that there cannot be a crowd within for Block 1, since participants had only made a single judgement for each trial at this point in the experiment.

##### Between Crowds

To test the conventional wisdom of the crowd effect, we created crowds of two and three people by averaging the responses different individuals gave to the same trial. Our procedure selected individual participants to form a crowd, averaging their responses together for a run of the entire task. To ensure fair comparison to the within crowds, “between crowds” were created by randomly sampling responses from one participant per block (e.g., a between crowd’s response in Block 3 consists of the response one crowd member made in Block 1, another in Block 2, and the other in Block 3). We calculated every between crowd possible at size 2 and size 3 from our sample of 113 participants.^3^

#### Statistical Approach

While we have comprehensively tested our hypotheses, we have deviated from our analysis plans to test these hypotheses more appropriately.^4^ Our revised analytic approach begins with a one-way ANOVA to test whether individual performance changed over the three blocks (i.e., practice or familiarity effects). Then, we use a series of paired samples *t*-tests to compare individual performance in each block to that of the within crowds. Finally, we use one-sample *t*-tests to compare the performance of within crowds to that of between crowds. Greenhouse-Geisser and Welch corrections have been applied when necessary. Cohen’s *d* effect sizes are reported with 95% confidence intervals.

Statistical analyses were performed in *R* (R Core Team, 2022), using packages including *report* (Makowski et al., 2023), *rstatix 0.7.1* (Kassambara, 2022), *patchwork 1.3.0* (Pedersen, 2024), and *dplyr 1.1.4* (Wickham et al., 2023). Further analyses were performed in MATLAB 24.2.0 (MATLAB, 2024).

### Results

To summarise our main results (see Figure 1), there was no change to individual performance in each block (i.e., no practice or familiarity effects from doing the task three times), the wisdom of the crowd within offered significant improvements compared to individual performance in each block, the wisdom of the crowd was larger for between crowds than within crowds, and crowds of three exceeded crowds of two. The patterns are very similar between the two dependent variables (AUC, overall accuracy) and across Experiment 1 and Experiment 2.

**Figure 1.**
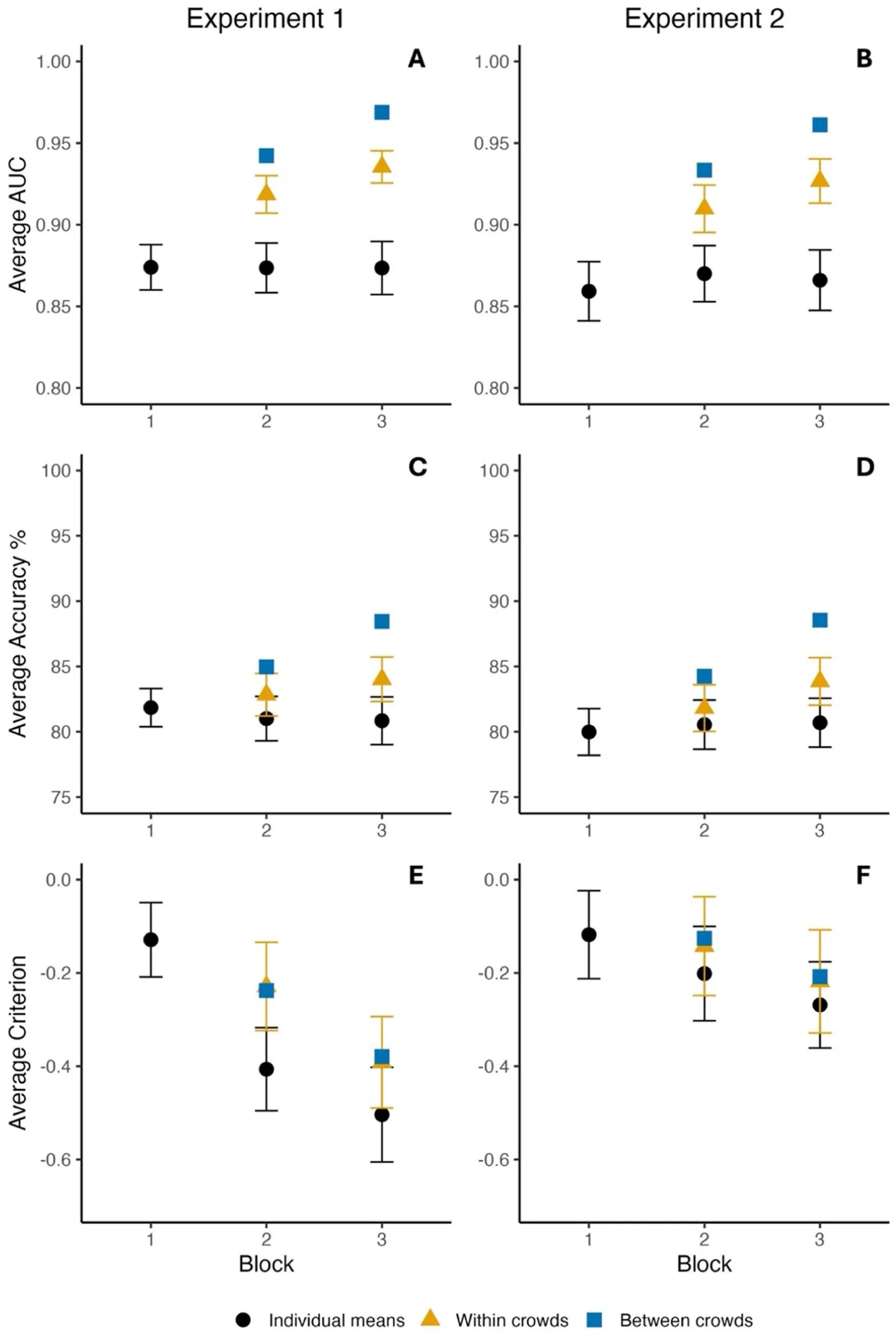
Measures of performance AUC (A, B), Overall Accuracy (C, D), and Criterion (E, F) in each crowd condition, plotted separately for Experiment 1 (left) and Experiment 2 (right). *Note.* The error bars are 95% CI. The between-crowd results (blue squares) are point estimates and, as such, have no error bars. AUC = area under the curve; CI = confidence interval.

#### Area Under the Curve

The AUC data are shown in Figure 1A. Individual performance was consistent across blocks, and a one-way ANOVA confirmed that there were no practice effects, *F*(1.71, 191.95) = 0.00, *p* = .992, η_*p*_^2^ = .00.

To test Hypothesis 1, we used paired samples *t*-tests to compare the performance of the within crowds to the individual data in Block 2 and Block 3. Within crowds significantly improved on individual performance in Block 2, *t*(112) = 12.87, *p* < .001, *d* = 1.21, 95% CI [0.97, 1.45], and Block 3, *t*(112) = 12.31, *p* < .001, *d* = 1.16 [0.92, 1.40], confirming the presence of the wisdom of the crowd within effect. Paired sample *t*-tests also showed that the within crowd at Block 2 had improved on the individual responses in Block 1, *t*(112) = 16.78, *p* < .001, *d* = 1.58 [1.30, 1.85], and that the within crowd of Block 3 exceeded that of Block 2, *t*(112) = 10.32, *p* < .001, *d* = 0.97 [0.75, 1.19]. A final paired samples *t*-test confirmed that the wisdom of the crowd within in Block 3 exceeded individual performance in Block 1, *t*(112) = 16.58, *p* < .001, *d* = 1.56 [1.28, 1.83]. These data support Hypothesis 1.

One sample *t*-tests confirmed that the wisdom of the crowd effect was significantly larger when the crowds consisted of different individuals (between crowds) compared to the same individual (within crowds), at both Block 2, *t*(112) = 4.15, *p* < .001, *d* = 0.39, 95% CI [0.20, 0.58] and Block 3, *t*(112) = 6.79, *p* < .001, *d* = 0.64 [0.43, 0.84], supporting Hypotheses 2 and 3. We cannot use inferential statistics to compare the performance of between crowds, since we computed every possible crowd. The average performance of between crowds simply is higher at Block 3 than at Block 2 (see Figure 1A).

#### Overall Accuracy

Our analysis of overall accuracy follows the same structure used for AUC, and foreshadowing the results, leads to the same conclusions. The descriptive statistics for overall accuracy are shown in Table 1. A one-way ANOVA showed that individual performance did not improve with practice across blocks, *F*(1.58, 176.65) = 1.74, *p* = .185, η_*p*_^2^ = .02 (see Figure 1C). Paired sample *t*-tests revealed that within crowds exceeded individual performance in Block 2, *t*(112) = 5.00, *p* < .001, *d* = 0.47, 95% CI [0.28, 0.66], and Block 3, *t*(112) = 8.25, *p* < .001, *d* = 0.78 [0.56, 0.98]. Further paired sample *t*-tests confirmed that the accuracy of within crowds at Block 2 exceeded individual responses in Block 1, *t*(112) = 2.45, *p* = .016, *d* = 0.23 [0.04, 0.42], and that within crowds at Block 3 exceeded those at Block 2, *t*(112) = 3.13, *p* = .002, *d* = 0.29 [0.11, 0.48]. A final paired sample *t*-test confirmed that the wisdom of the crowd within at Block 3 exceeded individual performance in Block 1, *t*(112) = 3.83, *p* < .001, *d* = 0.36 [0.17, 0.55]. These results support Hypothesis 1. One sample *t*-tests confirmed that the wisdom of the crowd effect was significantly larger for between crowds than within crowds, both at Block 2, *t*(112) = 2.61, *p* = .010, *d* = 0.25 [0.06, 0.43], and Block 3, *t*(112) = 5.23, *p* < .001, *d* = 0.49 [0.30, 0.69], supporting Hypotheses 2 and 3.

**Table 1.** Descriptive Statistics for Accuracy in Experiment 1.

Crowd	Block	Overall accuracy		Match trials		Mismatch trials	
		Mean (*SD*)	Range	Mean (*SD*)	Range	Mean (*SD*)	Range
Individual	1	81.8 (7.8)	57.5–98.8	85.0 (11.0)	47.5–100	78.7 (14.3)	32.5–100
Individual	2	81.0 (9.0)	56.2–97.5	90.3 (10.2)	52.5–100	71.7 (18.3)	12.5–100
Individual	3	80.8 (9.7)	57.5–98.8	92.2 (9.5)	60.0–100	69.5 (21.3)	15.0–100
Within	2	82.8 (8.7)	55.0–98.8	88.1 (11.7)	37.5–100	77.6 (16.9)	25.0–100
Within	3	84.0 (9.0)	56.2–100	92.2 (10.0)	55.0–100	75.8 (19.0)	12.5–100
Between	2	84.9 (6.8)	57.5–100	89.9 (9.2)	37.5–100	80.0 (13.9)	15.0–100
Between	3	88.5 (6.2)	51.2–100	95.2 (5.7)	40.0–100	81.7 (13.3)	2.5–100

*Note. SD* = standard deviation.

#### Criterion

There was a significant change in criterion across individual blocks, *F*(1.62, 181.40) = 74.65, *p* < .001, η_*p*_^2^ = .40 (see Figure 1E). Participants started with a liberal criterion in Block 1 (*M* = −0.13, *SD* = 0.42), which became stronger in Block 2 (*M* = −0.41, *SD* = 0.47) and Block 3 (*M* = −0.50, *SD* = 0.54). Paired samples *t*-tests showed that the liberal criterion was significantly larger for individuals than the within crowds in Block 2, *t*(112) = 8.06, *p* < .001, *d* = 0.76, 95% CI [0.55, 0.97], and Block 3, *t*(112) = 5.33, *p* < .001, *d* = 0.50 [0.30, 0.70]. Nonetheless, criterion did become more liberal over time for within crowds, such that within crowds of Block 2 had a stronger bias than individual responses in Block 1, *t*(112) = 4.42, *p* < .001, *d* = 0.42 [0.22, 0.61], and within crowds had a stronger bias in Block 3 than Block 2, *t*(112) = 8.54, *p* < .001, *d* = 0.80 [0.59, 1.01]. Criterion did not differ between within crowds and between crowds at Block 2, *t*(112) = 0.20, *p* = .840, *d* = 0.02 [−0.17, 0.20], or Block 3, *t*(112) = −0.25, *p* = .801, *d* = −0.02 [−0.21, 0.16].

#### Exploratory Analyses

##### Response Consistency

We investigated the consistency of individual responses across blocks. For this analysis, we look at the most conservative data, which is simply whether the participant made the correct identification decision in a trial, regardless of their confidence. This analysis revealed that 79.2% of match trials were answered consistently (i.e., correct 0/3 or 3/3 times), while participants changed their identification decision on 20.8% of trials (i.e., correct 1/3 or 2/3 times). It was a similar story for mismatch trials, with participants giving consistent responses to 63.9% of trials, and changing their decisions on 36.1% of trials. Figure 2A shows that the vast majority of consistent responses were three correct decisions, with less than 10% of trials answered incorrectly all three times. The wisdom of the crowd within effect can only emerge when participants give inconsistent responses to the same trial. Because participants are more likely to answer a trial correctly 2/3 times than 1/3 times, averaging their responses leads to improved performance. This pattern of response consistency is very similar to that reported by Bindemann et al. (2012).

**Figure 2.**
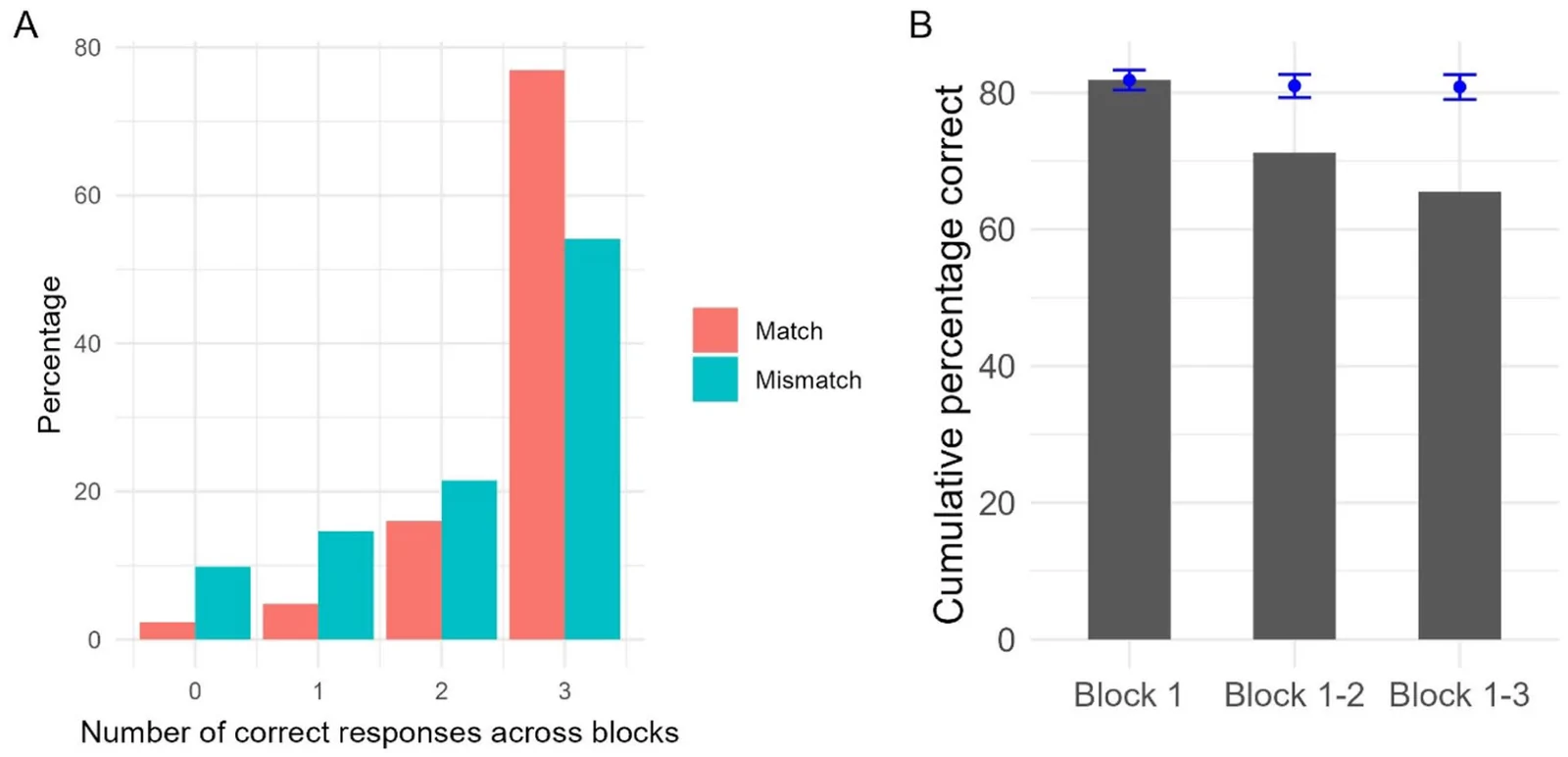
(A) Consistency of identification responses over the three blocks, (B) Cumulative accuracy across the blocks (bars), with individual block accuracy shown in blue points with 95% CI error bars.

##### Cumulative Accuracy

We investigated cumulative accuracy across the three blocks. That is, how many of the trials that were answered correctly in Block 1 were also answered correctly in Block 2, and then again in Block 3. Though mean overall accuracy was very similar in each individual block, Figure 2B shows that cumulative accuracy declined over the three blocks. After answering 81.8% of all trials correctly in Block 1, participants had only answered 71.2% of all trials correctly in both Block 1 and Block 2, and just 65.5% of trials in all three blocks. These data demonstrate that even though average individual accuracy was highly stable across blocks, participants achieved this consistency by answering different trials correctly each time. Again, this pattern of response consistency is very similar to that reported by Bindemann et al. (2012).

This overarching pattern is reflected in the correlation between an individual’s responses across blocks. Here we calculated the correlation between every individual’s responses (1–6) in two blocks, and then reported the average correlation across all participants. The average correlations were moderate-strong and positive: Blocks 1 and 2 (*M* = 0.69, 95%CI [0.64, 0.74]), Blocks 2 and 3 (*M* = 0.75 [0.70, 0.79]), and Blocks 1 and 3 (*M* = 0.68 [0.62, 0.73]). To complete this analysis, we also investigated the average correlation between the responses given by two different individuals to the same trials (i.e., between crowds). As expected, the average correlation between the responses of different participants across blocks is lower: Blocks 1 and 2 (*M* = 0.54 [0.51, 0.56]), Blocks 2 and 3 (*M* = .54 [0.52, 0.57]), and Blocks 1 and 3 (*M* = 0.54 [0.52, 0.56]).

##### Item Analysis

Finally, we investigated whether the wisdom of the crowd effect benefitted all face pairs (items) equally. We looked at the average change in accuracy for the item when answered by a crowd of 3, compared to the average accuracy for the same item in Block 1 (individual responses). Due to the difference in match and mismatch trial performance (see Table 1), we have plotted these items separately. The patterns of item accuracy change among within crowds (Figure 3A) are similar to those between crowds (Figure 3B). In both cases, identity match trials gained more in a crowd than mismatch trials. The interpretation of these figures is made more difficult by the shift in criterion over the task (see Figure 1E). Participants became more likely to respond “same” as the experiment went on, which is consistent with known time on task effects (Alenezi et al., 2015) and will increase match accuracy at the expense of mismatches. Nonetheless, there were identity-mismatch trials that improved among within crowds, although far more did so in between crowds. These figures also show the larger gains made by between crowds compared to within crowds. Figure 3C shows that the correlation between the gain an item experienced in a within crowd and in a between crowd was very high (*r* = .93, *p* < .001). These data suggest that within and between crowds offer improvements on the same trials.

**Figure 3.**
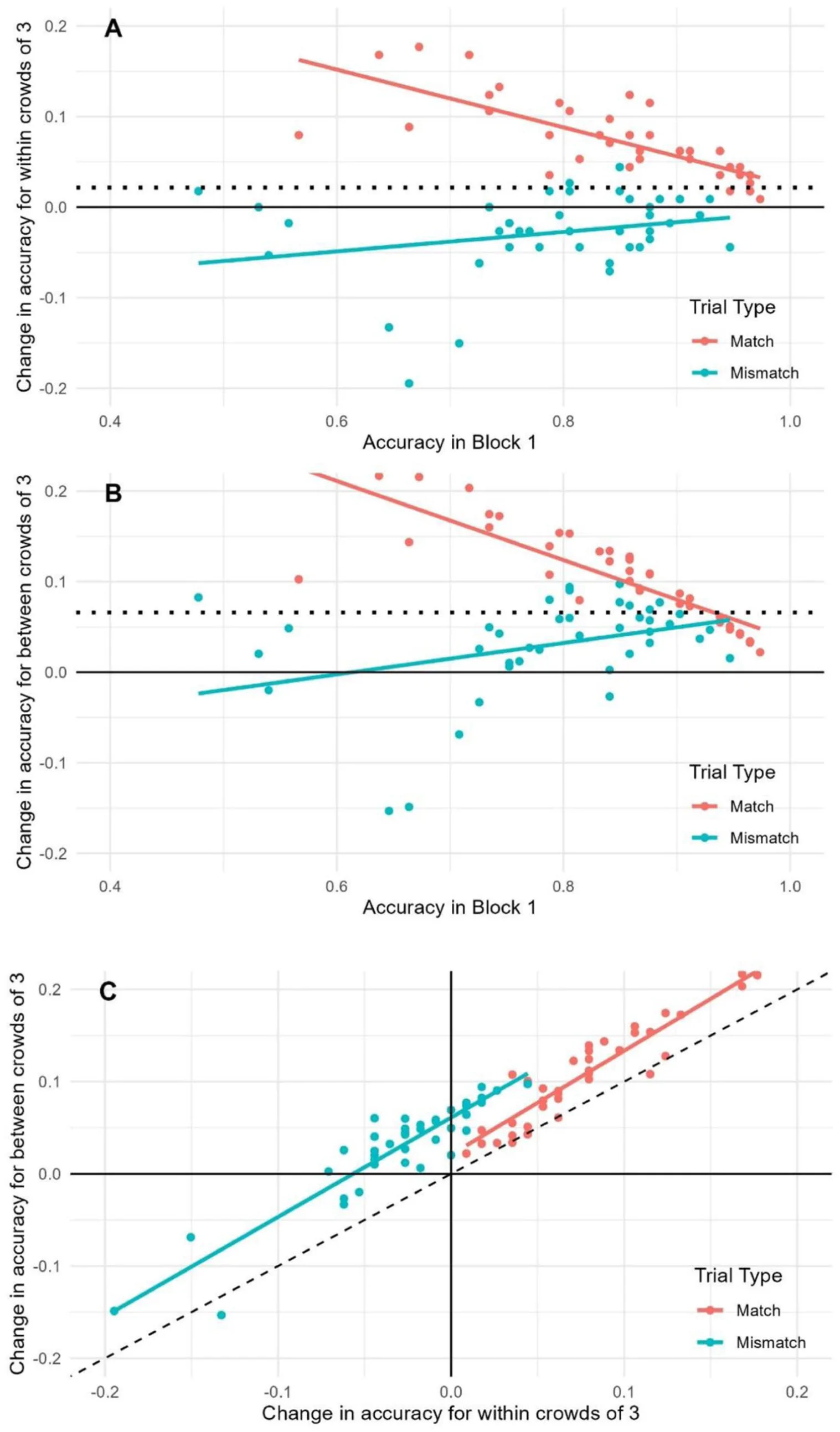
Experiment 1. Average accuracy change compared to baseline for items in (A) within crowds, (B) between crowds, and (C) the correlation of item gains across crowd type (within, between). The dotted line in (A) and (B) is the average change over all trials; in (C), it is the line of equality.

### Discussion

Our results show that the wisdom of the crowd within can improve unfamiliar face matching performance, even when participants are tested repeatedly in a single session. There was no evidence of practice effects; rather, improvements only occurred when an individual’s responses were aggregated to form a within crowd. Compared to individual performance in Block 1, the wisdom of the crowd within offered significant improvements (given here as percentage increase, not raw difference) in Block 2 (AUC = 5.0%; overall accuracy = 1.2%), which increased further by Block 3 (AUC = 7.0%; overall accuracy = 2.6%). In both cases, the improvement from Block 2 to Block 3 was also statistically significant. Finally, although within crowds significantly improved performance, between crowds formed by different observers made even larger gains compared to individual performance (Block 1) at both Block 2 (AUC = 7.8%; overall accuracy = 3.8%) and Block 3 (AUC = 10.9%; overall accuracy = 8.1%). These results support all three of our pre-registered hypotheses.

## Experiment 2

In Experiment 1, participants completed the same face matching task three times in a single session. Here, we investigate whether the wisdom of the crowd within becomes stronger if more time passes between repeated responses, an effect reported by Vul and Pashler (2008). Experiment 2 is identical to Experiment 1, except that the face matching task was completed once a week for 3 weeks. We offered the same three hypotheses supported in Experiment 1. We also made the new prediction **(H4)** that the wisdom of the crowd within effect would be larger in Experiment 2, when multiple days had passed between each block, than in Experiment 1.

### Method

#### Participants

Participants completed the GFMT2-S across three separate sessions, each approximately 1 week apart (minimum possible delay = 4 days, maximum possible delay = 10 days). Weekly recruitment details are given in Table 2. Our final sample for analysis consisted of 83 participants (*M*_age_ = 38.8, *SD* = 12.9; 46 female, 36 male, 1 non-binary) who completed all 3 sessions, passing all pre-registered exclusion criteria each time. Though this final sample fell below our pre-registered goal (*n* = 100), we did not complete additional data collection due to resource constraints.

**Table 2.** Details of Participant Recruitment Each Week in Experiment 2.

Week	Recruited	Final sample	Mean days between testing (*SD*, Min, Max)	Mean completion time (*SD*)	Payment in GBP (USD)
Week 1	122	118	N/A	11.12 min (5.13)	1.10 (1.36)
Week 2	98	92	7.54 (0.80, 6.91, 9.72)	10.30 min (5.55)	1.50 (1.86)
Week 3	85	83	6.82 (0.78, 4.88, 9.73)	9.78 min (5.78)	1.50 (1.86)

*Note.* Final sample is the number of participants who passed all exclusion criteria. Average completion time for all weeks is calculated from the 83 participants in our final sample. Payments increased after week 1 to ensure they exceeded Prolific’s payment guidelines. *SD* = standard deviation.

### Results

#### Area Under the Curve

Seen in Figure 1B, the results for AUC are very similar to Experiment 1. A one-way ANOVA showed that individual performance did not improve with practice across blocks, *F*(1.83, 149.77) = 1.22, *p* = .296, η_*p*_^2^ = .02. Paired samples *t*-tests showed within crowds outperformed individuals in Block 2, *t*(82) = 9.31, *p* < .001, *d* = 1.02, 95% CI [0.75, 1.29], and Block 3, *t*(82) = 12.67, *p* < .001, *d* = 1.39 [1.09, 1.69]. Paired samples *t*-tests also confirmed that within crowds at Block 2 outperformed individual responses in Block 1, *t*(82) = 10.64, *p* < .001, *d* = 1.17 [0.89, 1.45], and that the within crowds in Block 3 exceeded those in Block 2, *t*(82) = 8.80, *p* < .001, *d* = 0.97 [0.70, 1.22]. Finally, a paired sample *t*-test confirmed that the wisdom of the crowd within at Block 3 exceeded individual performance in Block 1, *t*(82) = 12.16, *p* < .001, *d* = 1.34 [1.04, 1.63]. These data support Hypothesis 1. One sample *t*-tests showed that the wisdom of the crowd was significantly larger for between crowds than within crowds in Block 2, *t*(82) = 2.78, *p* = .007, *d* = 0.31 [0.08, 0.52], and Block 3, *t*(82) = 4.37, *p* < .001, *d* = 0.48 [0.25, 0.71], supporting Hypotheses 2 and 3.

#### Overall Accuracy

Individual accuracy did not improve with practice across blocks, *F*(2, 164) = 0.50, *p* = .610, η_*p*_^2^ = .01 (see Figure 1D). Paired samples *t*-tests showed within crowds were more accurate than individuals in Block 2, *t*(82) = 2.28, *p* = .025, *d* = 0.25, 95%CI [0.03, 0.47], and Block 3, *t*(82) = 5.37, *p* < .001, *d* = 0.59 [0.35, 0.82]. Paired samples *t*-tests also confirmed that the within crowds of Block 2 outperformed individual responses at Block 1, *t*(82) = 3.99, *p* < .001, *d* = 0.44 [0.21, 0.66], but were themselves exceeded by the within crowds of Block 3, *t*(82) = 5.45, *p* < .001, *d* = 0.60 [0.36, 0.83]. Again, a paired sample *t*-test confirmed that the wisdom of the crowd within at Block 3 exceeded individual performance in Block 1, *t*(82) = 7.71, *p* < .001, *d* = 0.85 [0.59, 1.10]. These data support Hypothesis 1. One sample *t*-tests confirmed that between crowds outperformed within crowds at both Block 2, *t*(82) = 2.34, *p* = .022, *d* = 0.26 [0.04, 0.47] and Block 3, *t*(82) = 4.42, *p* < .001, *d* = 0.49 [0.26, 0.71], supporting Hypotheses 2 and 3.

#### Criterion

Criterion changed across individual blocks, *F*(2, 164) = 6.42, *p* = .002, η_*p*_^2^ = .07 (see Figure 1F). Participants started with a liberal criterion in Block 1 (*M* = −0.12, *SD* = 0.50), which increased in Block 2 (*M* = −0.20, *SD* = 0.54) and Block 3 (*M* = −0.27, *SD* = 0.49). Paired samples *t*-tests showed that the liberal criterion was significantly larger for individuals than the within crowds in Block 2, *t*(82) = 2.37, *p* = .020, *d* = 0.26, 95% CI [0.04, 0.48], but not in Block 3, *t*(82) = 1.61, *p* = .111, *d* = 0.18 [−0.04, 0.39]. Nonetheless, criterion did become more liberal over time for within crowds, but only at Block 3. Within crowds of Block 2 did not differ in bias from individual responses in Block 1, *t*(82) = 0.83, *p* = .408, *d* = 0.09 [−0.12, 0.31], but within crowds had a stronger bias in Block 3 than Block 2, *t*(82) = 3.77, *p* < .001, *d* = 0.41 [0.19, 0.64]. Criterion did not differ across within crowds and between crowds at Block 2, *t*(82) = 0.22, *p* = .826, *d* = −0.02 [−0.24, 0.19], or Block 3, *t*(82) = −0.16, *p* = .875, *d* = −0.02 [−0.23, 0.20].

#### Exploratory Analysis

As in Experiment 1, we investigated the average correlation between an individual’s responses (1–6) to each trial across blocks. Again, the average response correlation between blocks was moderate-strong and positive: Blocks 1 and 2 (*M* = 0.65, 95% CI [0.58, 0.71]), Blocks 2 and 3 (*M* = 0.68 [0.61, 0.74]), and Blocks 1 and 3 (*M* = 0.65 [0.57, 0.71]). We also calculated the average correlation between the responses given by two different people to the same trial (between crowds). As before, the response correlation between different participants was lower: Blocks 1 and 2 (*M* = 0.51 [0.48, 0.55]), Blocks 2 and 3 (*M* = 0.53 [0.49, 0.56]), and Blocks 1 and 3 (*M* = 0.51 [0.48, 0.55]).

##### Item Analysis

Items experienced a larger accuracy gain in a between crowd (Figure 4B) than a within crowd (Figure 4A), though the overall pattern of increase was similar regardless of crowd type. Match trials still experienced a larger improvement than mismatch trials. Compared to Experiment 1, more mismatch trials experienced a gain in within crowds. This result could be due to the smaller criterion shift that occurred in Experiment 2 – participants did not end the task with such a strong bias to respond “same,” which contributed to better accuracy on mismatch trials generally. Once again, there was a very strong correlation (*r* = .86, *p* < .001) between the improvement the items experienced in within and between crowds.

**Figure 4.**
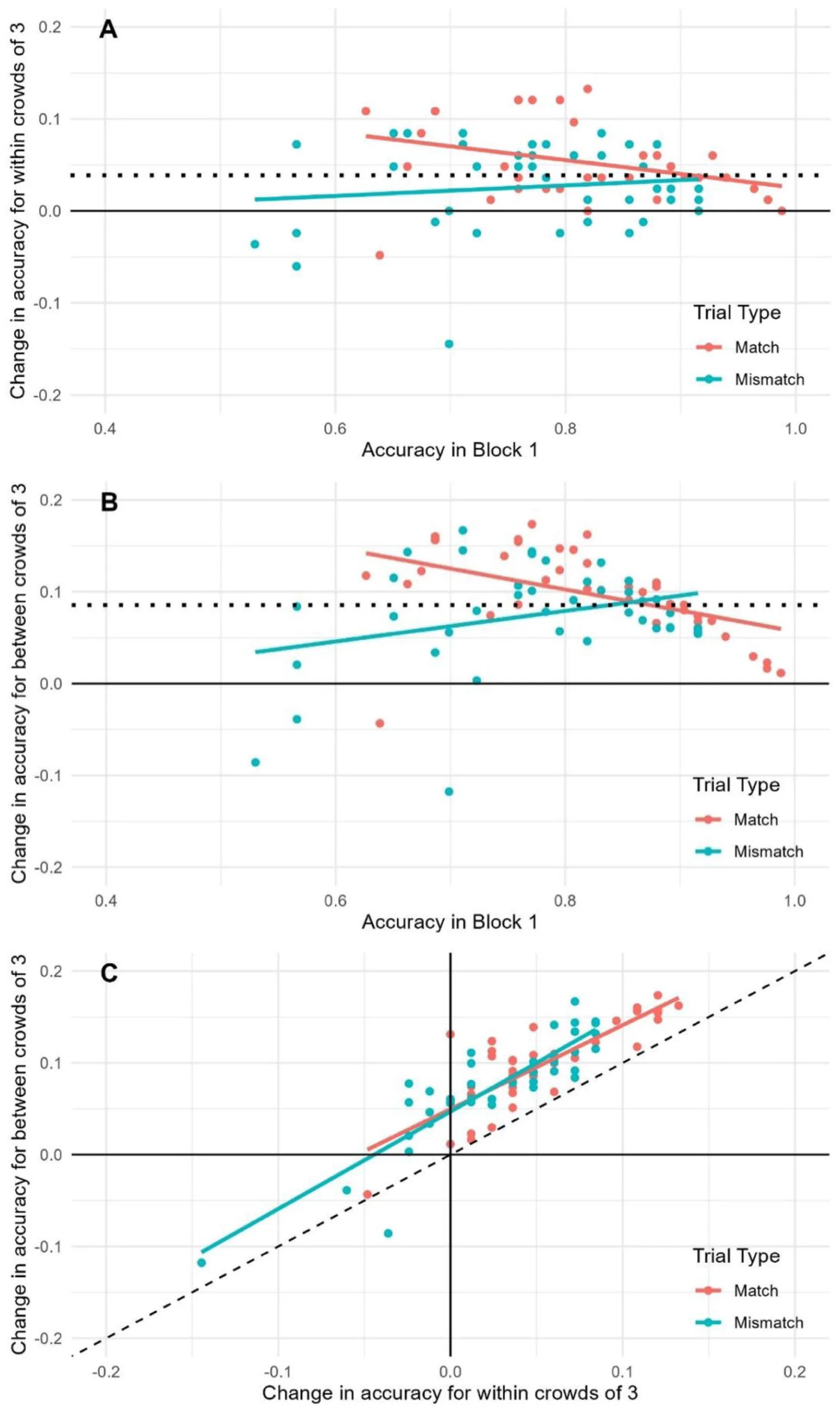
Experiment 2. Average accuracy change compared to baseline for items in (A) within crowds (B) between crowds, and (C) the correlation of item gains across crowd type (within, between). The dotted line in (A) and (B) is the average change over all trials; in (C), it is the line of equality.

#### Cross-Experiment Comparisons

Our final hypothesis predicted that the wisdom of the crowd within effect would be larger in Experiment 2, when more time had passed between decisions, than in Experiment 1. As such, we do not examine the performance of the between crowds here. Reported in our Supplemental Material, two initial ANOVAs (AUC, overall accuracy) comparing individual performance in each Block (1, 2, 3) between Experiments (1, 2) returned no significant effects, indicating that individual performance was very consistent across blocks, regardless of the delay between task repetitions (which is consistent with visual inspection of Figure 1).

##### Area Under the Curve

An ANOVA with Block (1, 2, 3) and Experiment (1, 2) as factors and AUC of the within crowds as the dependent variable returned a significant main effect of Block, *F*(1.19, 231.56) = 363.32, *p* < .001, η_*p*_^2^ = .65, which confirmed the presence of the wisdom of the crowd within effect. The main effect of Experiment was not significant, *F*(1, 194) = 1.15, *p* = .285, η_*p*_^2^ = .01. The critical interaction between Block and Experiment was also non-significant, *F*(1.19, 231.56) = 0.98, *p* = .337, η_*p*_^2^ = .01. The wisdom of the crowd within effect was a similar size regardless of whether participants made repeated responses in the same session or over multiple days.

##### Overall Accuracy

As above, an ANOVA with block and experiment as factors and overall accuracy as the dependent variable returned a significant main effect of block, *F*(1.66, 322.07) = 42.39, *p* < .001, η_*p*_^2^ = .18, while the main effect of experiment was non-significant, *F*(1, 194) = 0.67, *p* = .413, η_*p*_^2^ < .01. Unlike our analysis of AUC, however, there was a small, significant interaction between the two factors, *F*(1.66, 322.07) = 3.36, *p* = .045, η_*p*_^2^ = .02. We used post hoc *t*-tests to investigate the significant interaction, comparing the accuracy of within crowds from each experiment at all three blocks; however, none of the comparisons were statistically significant (see Supplemental Material).

Our interpretation of this result is that the marginal interaction likely stems from individual accuracy being slightly, but not significantly, lower in Block 1 for participants in Experiment 2 (*M* = 80.0, *SD* = 9.5) compared to those in Experiment 1 (*M* = 81.8, *SD* = 7.8), whereas the performance of the within crowds in Blocks 2 and 3 was similar between experiments (see Tables 1 and 3). Reported in our Supplemental Material, further analysis of these data as change scores (to account for any initial baseline differences) suggests that the increase in overall accuracy was slightly larger with the delay, but only in Block 3. But since this effect is only marginal and not reflected in our primary performance measure of AUC, we conclude that the wisdom of the crowd within effect was no larger with a greater delay between repeated decisions, refuting our final hypothesis.

**Table 3.** Descriptive Statistics for Accuracy in Experiment 2.

Crowd	Block	Overall accuracy		Match trials		Mismatch trials	
		Mean (*SD*)	Range	Mean (*SD*)	Range	Mean (*SD*)	Range
Individual	B1	80.0 (9.5)	52.5–96.3	82.7 (14.3)	42.5–100	77.3 (17.4)	10.0–100
Individual	B2	80.5 (10.0)	50.0–98.8	85.2 (14.8)	35.0–100	75.9 (18.5)	5.0–100
Individual	B3	80.7 (9.9)	53.7–98.8	86.8 (13.3)	50.0–100	74.6 (17.9)	17.5–100
Within	B2	81.8 (9.5)	51.2–98.8	84.5 (15.0)	40.0–100	79.1 (17.8)	2.5–100
Within	B3	83.9 (9.7)	52.5–100	87.8 (15.0)	37.5–100	79.9 (18.1)	5.0–100
Between	B2	84.3 (7.3)	53.7–100	87.0 (11.0)	25.0–100	81.5 (14.1)	15.0–100
Between	B3	88.5 (6.1)	48.7–100	92.3 (7.8)	27.5–100	84.8 (11.9)	7.5–100

*Note. SD* = standard deviation.

##### Criterion

Unlike the other measures, a mixed measures ANOVA showed that criterion differed for individual blocks between experiments, returning significant main effects of Block, *F*(1.79, 346.45) = 53.89, *p* < .001, η_*p*_^2^ = .22, and Experiment, *F*(1, 194) = 5.38, *p* = .002, η_*p*_^2^ = .03, as well as a significant interaction between the two factors, *F*(1.79, 346.45) = 11.06, *p* < .001, η_*p*_^2^ = .05. Independent samples *t*-tests showed that criterion did not differ between experiments at Block 1, *t*(158.65) = 0.16, *p* = .874, *d* = 0.02, 95% CI [−0.26, 0.31], but that Experiment 1 showed a stronger liberal criterion than Experiment 2 in Block 2, *t*(163.32) = 2.77, *p* = .006, *d* = 0.40 [0.11, 0.69], and Block 3, *t*(185.19) = 3.18, *p* = .002, *d* = 0.46 [0.17, 0.74].

A similar pattern was evident in the cross-experiment comparison of criterion among within crowds. The main effect of Block was significant, *F*(1.57, 304.56) = 48.75, *p* < .001, η_*p*_^2^ = .20, though the main effect of Experiment was not, *F*(1, 194) = 1.61, *p* = .206, η_*p*_^2^ = .01. Nonetheless, the interaction between the two factors was significant, *F*(1.57, 304.56) = 9.47, *p* < .001, η_*p*_^2^ = .05. Independent sample *t*-tests showed that criterion did not differ between experiments at Block 1, *t*(158.65) = 0.16, *p* = .874, *d* = 0.02, 95% CI [−0.26, 0.31], or Block 2, *t*(164.90) = 1.11, *p* = .269, *d* = 0.16 [−0.12, 0.45]. For Block 3, however, the criterion was significantly more liberal in Experiment 1 than Experiment 2, *t*(164.00) = 2.14, *p* = .034, *d* = 0.31 [0.02, 0.60]. These data show that the week delay between repetitions suppressed, but did not stop, the liberal response bias drift towards “match” responses.

##### Response Correlations

Finally, we compared the average correlation between individuals’ responses in Experiment 1 and Experiment 2, to explore the effect of the delay on the wisdom of the crowd within. Between Experiments 1 and 2, the average correlations between an individual’s responses did not differ between Blocks 1 and 2 (*t*[177.41] = 1.51, *p* = .133) or Blocks 1 and 3 (*t*[175.41] = 1.21, *p* = .230). The average response correlation between Blocks 2 and 3 was larger in Experiment 1 (*M* = 0.75, *SD* = 0.16) than Experiment 2 (*M* = 0.68, *SD* = 0.19, *t*[181.35] = 2.69, *p* = .008), suggesting that responses were slightly less consistent between Blocks 2 and 3 when more time had passed.

### Discussion

The findings of Experiment 2 directly replicate those of Experiment 1. Compared to individual performance in Block 1, the wisdom of the crowd within offered a significant improvement by Block 2 (AUC = 5.9%; overall accuracy = 2.3%), which continued to increase by Block 3 (AUC = 7.9%; overall accuracy = 4.8%). These improvements were slightly larger than Experiment 1 (by approximately 1%–2%), but not significantly so, as our cross-experiment comparisons were non-significant. A delay in making repeated decisions did not result in a significantly larger wisdom of the crowd within effect. Although this result was surprising based on our initial expectation of replicating Vul and Pashler (2008), it is consistent with Kramer et al.’s (2023) findings in an unfamiliar face matching task. The delay between task repetitions did, however, have a significant effect on criterion, with those in the week delay condition showing a less severe drift towards a liberal “same” response bias than participants in Experiment 1. Once again, crowds offered a larger improvement when they consisted of different people, at Block 2 (AUC = 8.6%; overall accuracy = 5.3%) and Block 3 (AUC = 11.9%; overall accuracy = 10.7%).

## General Discussion

Across two experiments, we show that asking the same participant to evaluate the same face pairs multiple times can improve their overall performance in an unfamiliar face matching task, via a “wisdom of the crowd within” effect. The effect was evident and statistically significant after two decisions, and became larger when all three of their decisions were combined. Though significant improvement occurred via the wisdom of the crowd within, larger gains were made when crowds consisted of different individuals. These first three results were replicated across both experiments and supported our first three hypotheses. However, contrary to our final prediction, allowing more time to pass between repetitions of the task (multiple days, as opposed to several minutes in the same testing session) did not lead to a significantly larger wisdom of the crowd within effect. Taken together, our findings show that the wisdom of the crowd within could be a viable method to improve an individual’s unfamiliar face matching performance. Such an effect can be established within a single testing session, without the need for a delay between repeated decisions.

We found that the wisdom of the crowd within effect was present after two decisions, replicating Kramer et al. (2023). The similarity in the effect is notable, with Kramer et al. (2023) reporting an increase in AUC of approximately 5% after two decisions, and our data showing a 5% to 6% increase. But Kramer et al. (2023) only asked their participants to complete the same task twice. We show that a third decision continues to add to the wisdom of the crowd within effect, with AUC increasing further to 7% to 8% above individual performance in Block 1. The benefit of this third decision indicates that participants are continuing to reduce random error in their responses. However, it also appears that the increase from two decisions to three may be smaller than the increase from one to two. Such diminishing returns would be consistent with the conventional wisdom of the crowd effect, when large performance gains occur initially by adding individuals to small crowds, whereas adding individuals to already large crowds has little effect (White et al., 2013). For example, Carragher and Hancock (2024) reported that crowds comprising 40 individuals improved overall face matching performance by 21%, but that half of this improvement had already occurred among crowds of just 4 individuals. Diminishing returns in the wisdom of the crowd within were also reported by Rauhut and Lorenz (2011), who noted that repeated responses from the same individual contained less variability than crowds of different people. Our analysis of average response correlation showed a similar effect. Repeated responses made by the same participant were more strongly correlated than the responses of two different participants. Nonetheless, further research may investigate whether there is any additional benefit to asking the same individual to provide more than three decisions for the wisdom of the crowd within in an unfamiliar face matching task.

Both of our experiments found that aggregating decisions from different observers (between crowds) led to significantly larger increases in accuracy than creating crowds from the same person (within crowds). Vul and Pashler (2008) found the same effect in a general knowledge task, which the authors attributed to there being greater independence between the decisions provided by two different people, compared to two decisions from the same person. This effect was also reported by Rauhut and Lorenz (2011) in their general knowledge task. Their results showed that while within crowds offered an improvement, asking just one other person would always lead to larger improvements than repeatedly asking the same person. Kramer et al. (2023), however, did not find a significant difference between crowd types (between, within) in their face matching task. This discrepancy may be due to differences in statistical power between our two studies. We pre-registered recruiting larger samples of participants for appropriate power to detect smaller effects, whereas Kramer et al. (2023) report recruiting a sample appropriate for a medium-sized effect. Indeed, our results suggest that at crowd size 2, between crowds outperformed within crowds with a small-medium effect size (AUC: Cohen’s *d* = 0.31–0.39). Visual inspection of Figure 1 in Kramer et al. (2023) suggests that between crowds tended to outperform within crowds, but without reaching statistical significance (reported Cohen’s *d* = 0.21–0.22). In both experiments here, we found that between crowds exceeded within crowds of 3, but with larger effect sizes (Cohen’s *d* = 0.48–0.64), giving us confidence in our conclusion that crowds of different individuals outperform the crowd within.

A week’s delay between repeated responses did not increase the wisdom of the crowd within effect in our study. While a delay benefitted participants in Vul and Pashler’s (2008) general knowledge task, Kramer et al.’s (2023) figures suggest that a delay was not beneficial in their unfamiliar face matching task either. Several methodological differences may help to explain this discrepancy. First, Vul and Pashler (2008) asked participants general knowledge questions that required exact numeric responses (i.e., “What percentage of the world’s airports are in the United States?”, p. 645), whereas the unfamiliar face matching tasks required identification decisions on a scale (see below). Second, Vul and Pashler’s (2008) task consisted of 8 questions, whereas the unfamiliar face matching tasks had 40 (Kramer et al., 2023) or 80 trials (current study). A delay may have benefitted Vul and Pashler’s (2008) participants simply because they may have remembered their previous responses when asked again immediately, but were less likely to remember 3 weeks later. By contrast, the average correlation between an individual’s responses in adjacent blocks of our face matching task was already only moderate-strong (.69 to .75) in Experiment 1, and only slightly lower (.65–.68) with the delay in Experiment 2. This pattern suggests that most of the variability in an individual’s responses was already present when the repetitions occurred minutes apart, which may help to explain why a week’s delay did not offer significant additional benefit in our unfamiliar face matching task.

Importantly, the moderate-to-strong individual response correlations referenced above do not suggest that our participants were responding carelessly across repetitions of the task. Rather, Figure 1 shows that average individual accuracy was very similar across the three blocks in both of our experiments. This result replicates the patterns of response consistency reported by Bindemann et al. (2012), whereby participants maintained consistent average accuracy across task repetitions, but did so by answering a handful of trials differently each time. Our results potentially speak to Vul and Pashler’s (2008) original explanation for the wisdom of the crowd within effect itself – participants may change their responses to the same trial not because they are careless or inattentive, but because their responses are drawn from an internal probability distribution that can be influenced by many factors, including random noise. The correlations for item responses in the between crowds can also contribute to our understanding of the wisdom of the crowd effect, and why between crowds achieve larger gains than within crowds. The wisdom of the crowd effect depends on the independence of the decisions made by members of the crowd. While there is some variability in the responses made by the same person, there is more associated with decisions made by different people. Greater variability in responses means that errors are less correlated in between crowds than within crowds, meaning they are more likely to be overturned with inputs from other crowd members. This result explains why the conventional wisdom of the crowd effect exceeds that of the crowd within.

The wisdom of the crowd within increased overall accuracy. Yet, this improvement was not carried equally across trials. Accuracy for match trials was initially higher than for mismatch trials, which is expected for the short GFMT2 (Popovic et al., 2025; White et al., 2022). From here, the wisdom of the crowd within resulted in larger gains for match trials than mismatch trials. In Experiment 1, accuracy for all 40 match trials increased through the wisdom of the crowd within by Block 3, whereas improvement occurred for only 10 mismatch trials. However, this pattern is influenced by shifting response bias, in which participants become more likely to respond “match” as time on task increases (Alenezi et al., 2015). Our results showed that this response bias drift was strongest among the individual responses. By averaging the current response with those given previously, crowds moderated this criterion shift (both within and between crowds). The liberal response bias drift was muted in Experiment 2, which we attribute to the delay in task repetitions – participants likely did not experience three separate sessions as a “long” face matching task (Alenezi et al., 2015). With this reduced criterion drift, the item analysis of Experiment 2 showed a consistent crowd improvement for more mismatch items, though match trials still benefitted most. Despite this discussion of accuracy and response bias, it is important to remember that these crowd improvements were confirmed by AUC, a measure of sensitivity that is independent of response bias, in both experiments. Nonetheless, our results highlight the need to consider response bias and criterion when examining wisdom of the crowd effects.

The use of a multi-point response scale is an important factor in obtaining a wisdom of the crowd effect, particularly among small crowds, in this face matching paradigm. We used a 6-point scale on which participants could indicate their identification decision (same or different) and confidence (definitely, probably, and guess). Kramer et al. (2023) used a similar 5-point scale, but in which the centre value was a “don’t know” response. If participants only made a binary response, which is common in many face matching tasks (Burton et al., 2010; Carragher & Hancock, 2020; Fysh & Bindemann, 2018), then a crowd of two – whether within or between – cannot improve on individual performance (Carragher & Hancock, 2024). Either both responses agree, in which case nothing is gained by combining them, or they do not, in which case a stalemate occurs (that we resolve via a simulated coin toss). Thus, a multi-point response scale has two advantages in this context. First, it likely makes it harder for participants to remember exactly how they responded to the same trial previously. Second, it allows for the possibility that a crowd of two may make an improvement. That Kramer et al. (2023) and we, using multi-point scales, show crowds of two achieve significant performance improvements, indicates that such response changes are common (see Supplemental Material for more details). Further experiments would be needed to test the merits of different response options, such as additional scale points.

The wisdom of the crowd within can improve unfamiliar face matching performance. However, one must also consider the limitations of this approach before implementing such a strategy in any applied scenario. Our participants were novices recruited online in exchange for a small payment. Although our participants were not explicitly told that they would be completing the exact same task three times, Kramer et al. (2023) did tell their participants, suggesting that forewarning of repetition does not stop the effect from occurring. But future research is needed to test whether professionals who perform face matching regularly also experience the wisdom of the crowd within. While professional experience does not automatically lead to superior face matching performance (i.e., White et al., 2014), some professionals may have undertaken training that encourages the use of fixed strategies for comparing images (Towler et al., 2021a). Such strategies are implemented with the aim of producing consistent identification decisions. So, while professionals do not always exhibit superior performance, those who implement standardised comparison strategies may be more likely to reach the same conclusions on repeated trials, potentially reducing the variability in their responses that the wisdom of the crowd within effect requires.

Noted above, the face matching task in our experiment had 80 trials, while Kramer et al.’s (2023) task had 40 trials. With many trials, these tasks may be well suited to generating the wisdom of the crowd within effect, simply because participants are unlikely to remember their previous responses. We can speculate that the wisdom of the crowd within may become less effective as the number of trials in the task is reduced. In some applied scenarios, an individual might spend hours, or even days, comparing a single pair of faces. It is difficult to imagine that asking this individual to make identification decisions repeatedly for this same single face pair, even with a delay, would result in variable responses. However, this is only a speculative supposition, and future research may wish to test the idea directly – can the wisdom of the crowd within benefit a smaller (or even a single) set of face matching decisions? These two considerations mean that the wisdom of the crowd within effect may be of limited value in some applied contexts, but further research is needed.

We recruited our participants from *Prolific* (Palan & Schitter, 2018), a research participation website that is commonly used for cognitive psychology experiments. Though we prevented individuals from our previous experiments from enrolling in the current project, they may still have participated in other experiments on the platform that used the short GFMT2. While familiarity with identities makes face matching much easier (Bruce et al., 2001; Jenkins et al., 2011; Johnston & Edmonds, 2009), our data suggest that any prior exposure to the task had a minimal effect on participant performance. First, individual performance in Block 1 (Experiment 1: *M* = 81.8, *SD* = 7.8; Experiment 2: *M* = 80.0, *SD* = 9.5) was slightly lower than the normed accuracy Popovic et al. (2025) reported for the short GFMT2 on Prolific (*M* = 82.9, *SD* = 7.46), suggesting that our participants did not find the task any easier than expected. Moreover, compared to their individual performance in Block 1, average overall accuracy in Block 3 fell by 1.0% in Experiment 1 and rose by 0.7% in Experiment 2, showing that completing the task twice did not improve individual performance on the short GFMT2. Nonetheless, average overall accuracy on the short GFMT2 is quite high, relative to other face matching tasks (e.g., the short KFMT: Fysh & Bindemann, 2018). Would the wisdom of the crowd within effect occur if the face matching task were harder?

Our exploratory item analyses offer some clues. The wisdom of the crowd can only improve performance when the majority of individual responses are correct.^5^ The decision of the crowd will ultimately end up reflecting the individual responses that are given. The wisdom of the crowd will offer little benefit on the easiest trials, simply because individuals are likely to answer them correctly anyway. The effect is also likely to offer limited benefit for trials that are difficult to resolve and near chance-level accuracy, since the decision of the crowd will sit near the decision threshold for these difficult trials. Rather, the wisdom of the crowd should offer the most benefit on trials that are “moderately” difficult – that is, those that the majority of participants answer correctly, but many are likely to get wrong. Our item analysis in Figures 3 and 4 confirms that the biggest gains from crowds are for items that have a baseline accuracy around 70%. The picture is more confused for Experiment 1, in Figure 3, because of the strong shift in the criterion that causes mismatch items to get worse. These results suggest that the wisdom of the crowd may not be equally beneficial across all items or tasks.

Unfamiliar face matching is often described as being “surprisingly difficult” because the average individual makes errors in standard laboratory tasks, even though the conditions are generally quite favourable (Fysh & Bindemann, 2017). While training individuals to become better at unfamiliar face matching over hours or days has led to limited success (Towler et al., 2019), strategies leveraging combined decision-making have been relatively successful (Dowsett & Burton, 2015; Jeckeln et al., 2018; Ritchie et al., 2022). The wisdom of the crowd effect (Galton, 1907; Surowiecki, 2005) means that statistical crowds of novices achieve significant improvements in unfamiliar face matching performance compared to their average individual performance (Balsdon et al., 2018; Carragher & Hancock, 2024; Cavazos et al., 2023; Davis et al., 2019; Kramer & Javorková, 2025; White et al., 2013). Here, we have replicated previous research (Kramer et al., 2023) showing that the wisdom of the crowd effect can occur within a single observer who is asked to make the same face matching decision multiple times (Vul & Pashler, 2008). We have extended this work to show that averaging three decisions from the same observer leads to larger gains than two decisions, and that a delay of days between repetitions does not confer additional benefit. However, we also found that crowds are most beneficial when they consist of different individuals. Nonetheless, the wisdom of the crowd within effect offers performance gains that are similar in size or even slightly larger than those reported for some individual training approaches (Carragher et al., 2022; Towler et al., 2021b). While further research is needed to examine whether the wisdom of the crowd within can benefit specific applied contexts, our data suggest that in some cases, two out of three might not be so bad after all.

## Supplemental Material

sj-pdf-1-qjp-10.1177_17470218261452983 – Supplemental material for Two Out of Three Ain’t Bad: Aggregating Multiple Identification Decisions from the Same Observer Improves Unfamiliar Face Matching Performance Supplemental material, sj-pdf-1-qjp-10.1177_17470218261452983 for Two Out of Three Ain’t Bad: Aggregating Multiple Identification Decisions from the Same Observer Improves Unfamiliar Face Matching Performance by Daniel J. Carragher and Peter J. B. Hancock in Quarterly Journal of Experimental Psychology

## Data Availability

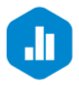 The data from the present experiment are publicly available at the Open Science Framework website: https://osf.io/wsv5f. Our Supplemental Material are available via the same link. The design, hypotheses, and analysis plan for both experiments were pre-registered prior to data collection: Experiment 1 (https://osf.io/tbgy3/overview) and Experiment 2 (https://osf.io/shw5u/overview).

## Notes

1. Binary responses are common in unfamiliar face matching tasks. They can allow for a wisdom of the crowd effect (using the majority rules criterion), except for when the crowd consists of two members (though any even-numbered crowds can return a split decision that does not improve performance either). In the current study, participants made their identification decisions on a scale from 1 to 6, which allowed us to test for improvement among crowds of two by averaging their numeric responses together.

2. Kramer et al. (2023) use the terms “inner” and “outer” crowds to refer to what we call “within” and “between” crowds, respectively. Both sets of terms exist in prior literature. We continue to use within and between as per our pre-registration.

3. We had planned to create the same number of between crowds as there were individual participants, but the random sampling of between crowds would produce another source of variation that complicates statistical inference. Instead, we computed every possible combination between crowds from our sample: *N* × (*N* − 1) (=12,656) of size 2, and *N* × (*N* − 1) × (*N* − 2) (=1,404,816) of size 3. We then treated the average performance of these between crowds as point estimates and used one-sample *t*-tests to compare their performance to that of the within crowds of the same size. Kramer et al. (2023) also report creating every possible combination between crowds for their analysis.

4. We had planned to use ANOVAs to compare crowd and individual performance in each block to address Hypotheses 2 and 3. However, we report *t*-tests instead, since the nature of these data violates ANOVA assumptions. Specifically, there can be no crowds at Block 1, so the data there would be the same in all three conditions. Similarly, the within crowd data at Blocks 2 and 3 are calculated including and therefore dependent on individual performance values from Block 1. Even with our revised analysis plan, comparing within crowds to individual performance violates the independence assumption of the *t*-tests. We therefore confirmed the comparisons using bootstrap resampling, which are reported in our Supplemental Material.

5. Though the inclusion of confidence in our response scale complicates this statement somewhat.
