# Supplementary material for: Two Out of Three Ain’t Bad: Aggregating Multiple Identification Decisions from the Same Observer Improves Unfamiliar Face Matching Performance
Source: Q J Exp Psychol (Hove). 2026 May 13;79(11):2960–78. doi: 10.1177/17470218261452983 (PMC13675558; doi:10.1177/17470218261452983)
Supplement: Supplementary file 1 [file sj-pdf-1-qjp-10.1177_17470218261452983.pdf]

Two out of three ain’t bad: Aggregating multiple identification decisions from the same observer improves unfamiliar face matching performance

*****SUPPLEMENTARY MATERIALS*****

Total Word Count: 2,300 (approx.)

Supplementary Results – Experiment 1

Bootstrapping

Using *t*-tests to compare the wisdom of the crowd within to individual performance, and between Blocks 2 and 3, violates independence assumptions. So, we used bootstrap resampling to provide an alternative means of comparison, computing 95% CI lower (LB) and upper (UB) bounds on the mean difference from 10,000 samples. For completeness, we use this approach to compare between crowds to within crowds at Block 2 and Block 3 also. In all cases, the lower bound is greater than 0, indicating a significant difference, supporting the conclusions reached in the main text.

Table S1

Bootstrap resampling of *t*-test comparisons from the main paper

Measure	Condition A	Block A	Condition B	Block B	Mean Difference	Lower Bound	Upper Bound
AUC	Within	2	Individual	2	0.0449	0.0386	0.0524
AUC	Within	3	Individual	3	0.0619	0.0529	0.0731
AUC	Within	2	Individual	1	0.0446	0.0395	0.0500
AUC	Within	3	Within	2	0.0169	0.0140	0.0204
AUC	Between	2	Within	2	0.0238	0.0136	0.0367
AUC	Between	3	Within	3	0.0334	0.0246	0.0440
Accuracy	Within	2	Individual	2	0.0183	0.0111	0.0252
Accuracy	Within	3	Individual	3	0.0317	0.0244	0.0398
Accuracy	Within	2	Individual	1	0.0098	0.0017	0.0175
Accuracy	Within	3	Within	2	0.0118	0.0046	0.0193
Accuracy	Between	2	Within	2	0.0213	0.0063	0.0380
Accuracy	Between	3	Within	3	0.0443	0.0287	0.0618

Signal Detection Measures

For readers more familiar with d' (sensitivity), Figure S1 is the equivalent of Figure 1 in the main text. Sensitivity was calculated according to the formula in Stanislaw and Todorov (1999). These figures are derived from the accuracy data, with values above 3.5 classified as a match response, and values of exactly 3.5 resolved with a simulated coin toss (see main text). The pattern for sensitivity shown in Figure S1C is consistent with the other performance measures (AUC, overall accuracy) reported in the main text. Within crowds exceed individual performance. Between crowds exceed within crowds. For both crowd types, the wisdom of the crowd effect is larger in Block 3 than Block 2.

Figure S1

Plots of supplementary performance measures: Optimal AUC Threshold (A, B), and Sensitivity (C, D) for Experiment 1 (Left) and Experiment 2 (Right)

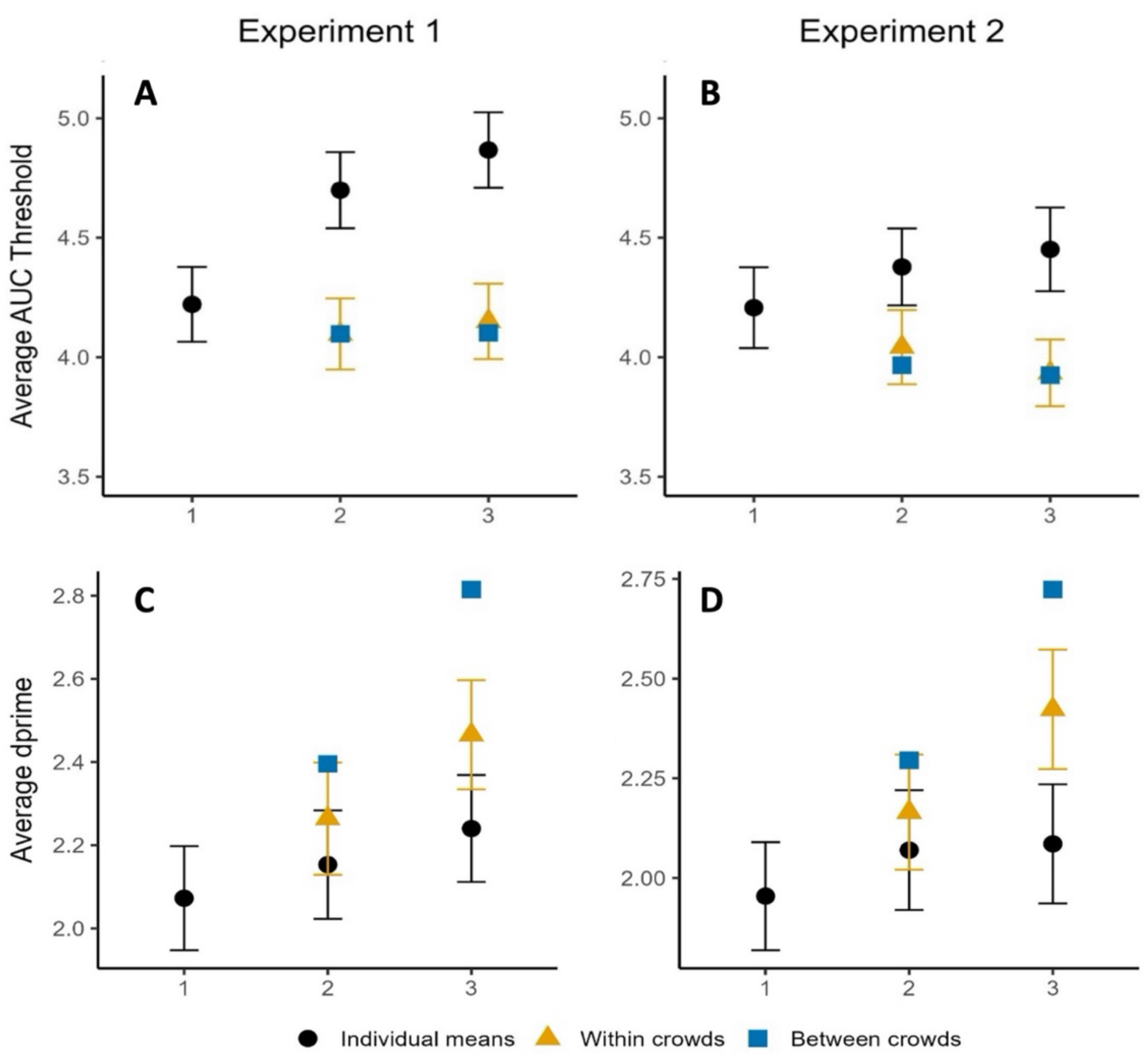

Note: The error bars are 95% CI. The between crowd results (blue squares) are point estimates and, as such, have no error bars.

Optimal AUC Thresholds

AUC is our primary measure of performance in the main text because participants responded to each trial on a scale from 1-6. This results in a distribution of responses for match and mismatch trials which, unless the participant is perfect, will overlap to some extent. The AUC procedure (*perfcurve* in MATLAB 24.2.0) also returns the optimum threshold for separation of match and mismatch trials[1]. Responses above the threshold are classified as match decisions. A low threshold will therefore do well on match trials at the cost of making some false matches, while a high threshold will correctly reject mismatches at the cost of missing some true matches. In an exploratory analysis, we examine the optimal threshold values, which are shown in the top panels of Figure S1. These optimal threshold values provide further insight into response biases, with values above 3.5 indicating a tendency to respond with "same" identification decisions.

It is apparent that individual thresholds rise with block, while those for both crowd types remain stable. A one-way ANOVA confirmed the rise in individual thresholds, $F(1.76, 197.46) = 34.70$, $p < .001$, $\eta_p^2 = .24$ (see Figure 1A). Paired *t*-tests confirmed that individual thresholds were greater than within crowd thresholds at Block 2, $t(112) = 8.27$, $p < .001$, $d = 0.78$, 95%CI[0.57, 0.99], and Block 3, $t(112) = 9.41$, $p < .001$, $d = 0.89$, 95%CI[0.67, 1.10]. However, paired *t*-tests confirmed that within crowd thresholds at Block 2 did not differ from Block 1, $t(112) = -1.70$, $p = .092$, $d = -0.16$, 95%CI[-0.35, 0.03], or from Block 3, $t(112) = 1.12$, $p = .265$, $d = 0.11$, 95%CI[-0.08, 0.29]. Finally, one sample *t*-tests confirmed no significant differences between thresholds for within and between crowds at Block 2, $t(112) = -0.07$, $p = .942$, $d = 0.00$, 95%CI[-0.19, 0.18], or Block 3, $t(112) = 0.40$, $p = .692$, $d = 0.04$, 95%CI[-0.15, 0.22].

[1] There may be more than one value for the optimal threshold. The *perfcurve* routine reports one of these. We explored reporting the median of all the possible optimum thresholds; this changed the average values slightly but not the pattern of results.

The rise in optimal threshold seen for individual responses is consistent with the liberal response bias drift seen for criterion in the main text, and relates to a significant rise in the overall average scale response, from 3.57 in Block 1, to 3.85 in Block 2, and 3.99 in Block 3, $F(1.46, 163.1) = 94.3$, $p < .001$, $\eta_p^2 = .46$. Falling criterion means that individuals are responding "same" more often – and here we see that their average response on the six-point scale also increases, suggesting these responses are becoming more confident. The AUC calculation responds by raising the threshold required for optimal separation of match and mismatch pairs.

On first inspection, these optimal threshold values may seem high: the midpoint of our 6-point scale is 3.5, yet the average values are above 4. This is a consequence of the computation, which will only test thresholds where there are responses (i.e., at integers for the individual responses). A threshold of 4 says classify anything at 4 or above as a match. For individual participants, a threshold of 4 is indicative of no bias, and higher values suggest a tendency to respond match more often. This mechanism also offers a possible reason as to why the crowd thresholds are lower than those for individuals. Averaging of crowd responses brings into play non-integer thresholds that were not available in the individual blocks. For example, an individual could only have an optimal threshold of 4 or 5, but a crowd of two can return a threshold of 4.5 (once their responses are averaged). Therefore, a lower optimal threshold for crowds may, in part, be due to their access to threshold values that were not available to individuals, rather than a profound difference in response behaviours. Although this alternative explanation may contribute to the patterns seen in our analysis of optimal threshold, the results are still consistent with our analysis of criterion, which are not subject to the same constraints, increasing our confidence that the effects seen in optimal AUC threshold are not entirely due to this calculation mechanism.

Transition Counts

In the main text we examine how participants change their decisions between blocks, but only counting instances where there was a change in accuracy (i.e., from correct in Block 1 to incorrect in Block 2). However, participants gave their responses on a scale from 1 (definitely different) to 6 (definitely same), so this binary analysis will miss response changes that do not cross the identification decision threshold (i.e., from "guess same" in Block 1 to "definitely same" in Block 2). Here we examine how responses changed between blocks along the entire scale. For ease of interpretation, we have transformed the meaning of the response scale for this analysis (by reverse coding the scale for mismatches), such that values of 1 now indicate a confidently incorrect response and 6 now means a confidently correct response (for both match and mismatch trials).

Figure S2 shows histograms of the size of the change, for match and mismatch trials separately, between Blocks 1 and 2, and Blocks 2 and 3. The maximum possible change is 5, either towards (positive values) or away (negative values) from a correct answer. The error bars are 95% CI from resampling the population 1000 times.

Figure S2

Histograms showing size of change towards a correct response, error bars are 95%CI.

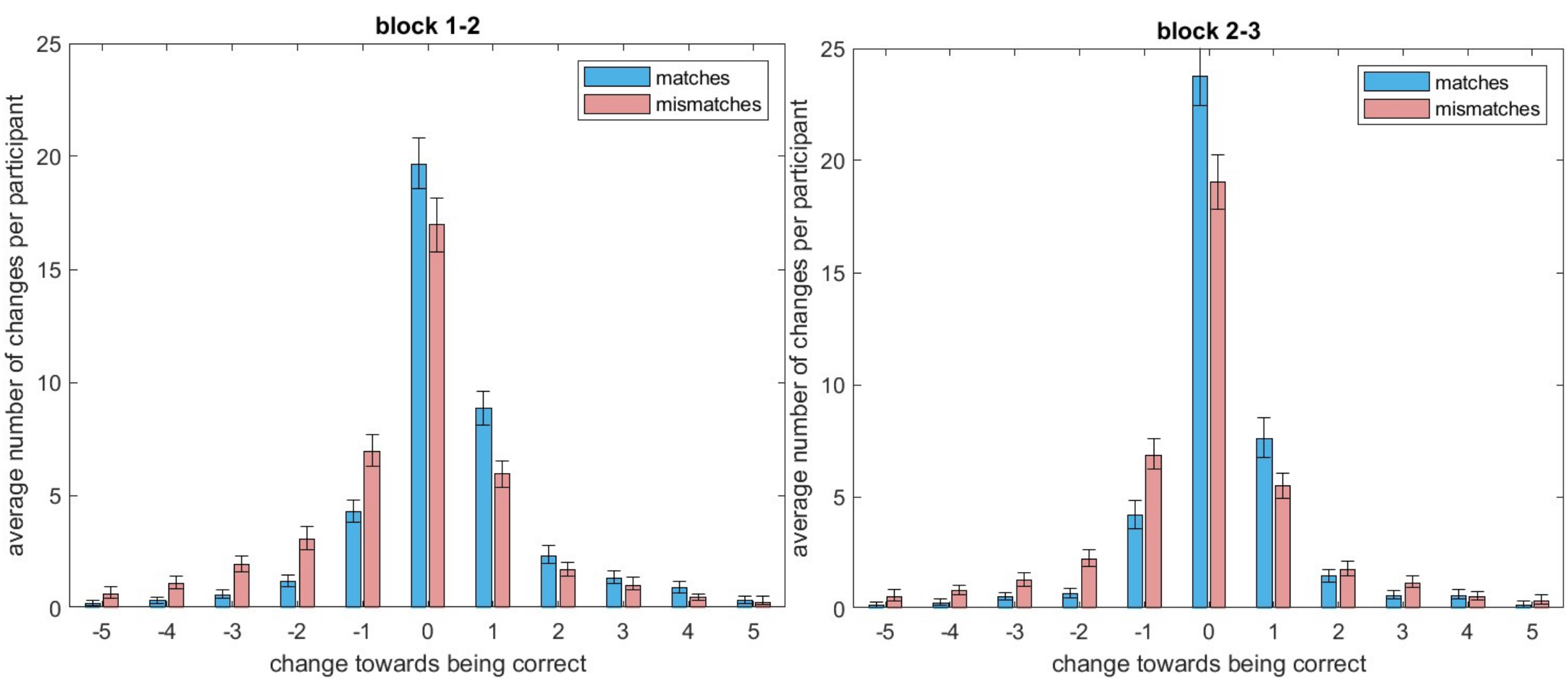

Tables S2-5 show these transitions in more detail, with each row showing the initial scale response and the next six columns showing the frequency of responses at each scale point in the next block. As above, the responses to mismatch items have been reversed, such that 1 means a confidently incorrect response while 6 is a confidently correct response. These are frequencies totalled across all participants in Experiment 1. So, for example, of all 157 confidently incorrect (1) responses to match trials in Block 1 (Table S2), 32 stayed with a confidently incorrect response (1) in Block 2, but 39 made the largest possible change to a confidently correct response (6). The next two columns show the total number of incorrect and correct responses, and the final column shows the percentage of correct responses. Of the 157 total confidently incorrect responses to match trials in Block 1, 65% were changed to a correct response in Block 2. However, among the 178 confidently incorrect responses to mismatches in Block 1 (Table S4), only 29.2% were changed to a correct response in Block 2.

Table S2

Match Trials: Block 1 to Block 2

From	To: 1	2	3	4	5	6	Incorrect	Correct	%correct
1	32	17	6	20	43	39	55	102	65
2	18	44	20	53	97	58	82	208	71.7
3	9	17	30	65	75	35	56	175	75.8
4	14	37	43	188	288	125	94	601	86.5
5	22	47	38	146	690	610	107	1446	93.1
6	22	15	7	51	261	1238	44	1550	97.2

Note: Values 1-3 always = incorrect, 4-6 always = correct.

Table S3

Match Trials: Block 2 to Block 3

From	To: 1	2	3	4	5	6	Incorrect	Correct	%correct
1	33	11	5	14	34	20	49	68	58.1
2	15	48	18	27	37	32	81	96	54.2
3	5	20	18	43	42	16	43	101	70.1
4	19	9	36	158	211	90	64	459	87.8
5	21	33	25	125	675	575	79	1375	94.6
6	18	10	8	37	277	1755	36	2069	98.3

Note: Values 1-3 always = incorrect, 4-6 always = correct.

Table S4

Mismatch Trials: Block 1 to Block 2

From	To: 1	2	3	4	5	6	Incorrect	Correct	%correct
1	85	28	13	10	13	29	126	52	29.2
2	54	121	57	33	65	40	232	138	37.3
3	43	84	80	95	74	39	207	208	50.1
4	28	62	113	182	180	74	203	436	68.2
5	53	128	129	200	457	311	310	968	75.7
6	70	70	61	112	334	993	201	1439	87.7

Note: Values 1-3 always = incorrect, 4-6 always = correct.

Table S5

Mismatch Trials: Block 2 to Block 3

From	To: 1	2	3	4	5	6	Incorrect	Correct	%correct
1	172	55	27	21	17	41	254	79	23.7
2	104	158	70	44	73	44	332	161	32.7
3	41	110	106	87	71	38	257	196	43.3
4	29	52	110	232	152	57	191	441	69.8
5	42	74	81	177	495	254	197	926	82.5
6	60	48	41	77	273	987	149	1337	90.0

Note: Values 1-3 always = incorrect, 4-6 always = correct.

Supplementary Results - Experiment 2

Signal Detection Measures

Figure S1D shows that the values for sensitivity are slightly lower in Experiment 2 than Experiment 1. However, this lower sensitivity seems mostly due to a lower starting point for individual performance in Block 1, an effect noted in the main text for the other performance measures. The patterns for sensitivity otherwise remain highly similar across the two experiments. Individual performance is exceeded by within crowds, which are exceeded by between crowds. Crowds of three outperform crowds of two. Together, these data are similar to those reported in the main text, suggesting that a delay does not lead to a larger wisdom of the crowd within effect.

Optimal AUC Thresholds

The summary results are shown in Figure S1B. A one-way ANOVA indicates that the rise in individual thresholds with Block is significant, $F(2, 164) = 3.10$, $p = .048$, $\eta_p^2 = .04$. Paired *t*-tests confirmed that thresholds were significantly higher for individuals than within crowds, both at Block 2, $t(82) = 5.47$, $p < .001$, $d = 0.60$, 95%CI[0.36, 0.83], and Block 3, $t(82) = 6.94$, $p < .001$, $d = 0.76$, 95%CI[0.52, 1.00]. Paired *t*-tests showed that within crowd thresholds at Block 2 were lower than Block 1, $t(82) = -2.04$, $p = .044$, $d = -0.22$, 95%CI[-0.44, -0.00], but that Block 2 did not differ from Block 3, $t(82) = -1.65$, $p = .103$, $d = -0.18$, 95%CI[-0.40, 0.04]. One sample *t*-tests confirmed no significant differences in threshold between the within and between crowds at Block 2, $t(82) = 0.85$, $p =.396$, $d = 0.09$, 95%CI[-0.12, 0.31] or Block 3, $t(82) = 0.28$, $p = .780$, $d = 0.03$, 95%CI[-0.18, 0.25].

Cross Experiment Comparisons – Additions from main text

Individual Performance

AUC. An ANOVA with Block (1, 2, 3) and Experiment (1, 2) as factors and the AUC of the individual participants as the dependant variable showed no effect of Block, $F(1.89, 366.20) = 0.75$, $p = .468$, $\eta_p^2 = .004$, or Experiment, $F(1, 194) = 0.55$, $p = .458$, $\eta_p^2 = .003$. The interaction between the two factors was also non-significant, $F(1.89, 366.20) = 0.86$, $p = .418$, $\eta_p^2 = .004$.

Overall Accuracy. The main effect of Block was non-significant, $F(1.81, 350.66) = 0.07$, $p = .922$, $\eta_p^2 = .00$, as was the main effect of Experiment, $F(1, 194) = 0.45$, $p = .503$, $\eta_p^2 = .002$. The interaction between the two factors was similarly non-significant, $F(1.81, 350.66) = 1.92$, $p = .153$, $\eta_p^2 = .010$.

Overall Accuracy – Significant Interaction

To interpret the significant interaction from the main text, we used paired sample *t*-tests to compare the within crowds from the two experiments in each block. These comparisons were all non-significant: Block 1, $t(154.94) = 1.46$, $p = .146$, $d = 0.21$, 95%CI[-0.07, 0.50]); Block 2, $t(167.43) = 0.77$, $p = .440$, $d = 0.11$, 95%CI[-0.17, 0.40]; Block 3, $t(169.76) = 0.12$, $p = .906$, $d = 0.02$, 95%CI[-0.27, 0.30]).

Change Scores

The initial individual performance of participants in Experiment 2 was slightly lower than Experiment 1 (see Figure 1, main text), which may complicate our comparison between experiments. As a final check, we undertook an exploratory analysis and calculated difference scores for each participant, subtracting their Block 1 performance from their performance in Block 2 and Block 3 to leave a measure of improvement – the wisdom of the crowd within – for fairer comparison between experiments.

AUC. Welch paired sample *t*-tests showed that these AUC change scores were not significantly different between the two experiments at Block 2, $t(131.85) = -1.10$, $p = .272$, $d = -0.19$, 95%CI[-0.53, 0.15]), or Block 3, $t(149.64) = -0.91$, $p = .366$, $d = -0.15$, 95%CI[-0.47, 0.17]), which is consistent with the non-significant full ANOVA in the main text.

Overall Accuracy. For overall accuracy change, the difference between the experiments was also non-significant at Block 2, $t(179.60) = -1.38$, $p = .170$, $d = -0.21$, 95%CI[-0.50, 0.09]). However, the paired *t*-tests showed a small significant difference at Block 3, with the wisdom of the crowd within larger in Experiment 2 (raw change 3.90%) than Experiment 1 (raw change 2.20%), $t(193.75) = 2.25$, $p = .026$, $d = 0.32$, 95%CI[0.04, 0.61]), which may help to explain the marginal interaction reported in the main text. Although this difference is consistent with our hypothesis, we believe the result is likely negligible, given the marginal nature of the significant interaction in the cross-experiment comparison ANOVA (main text), the small size of the effect itself, and the fact that the same comparisons were non-significant for our primary measure of AUC.

Optimal AUC Thresholds

Individual Performance. A 3 x 2 ANOVA comparing AUC thresholds in individual blocks between experiments returned a significant effect of Block, $F(2, 388) = 25.71$, $p < .001$, $\eta_p^2 = .12$, a significant effect of Experiment, $F(1, 194) = 5.46$, $p = .021$, $\eta_p^2 = .03$, and a significant interaction, $F(2, 388) = 4.98$, $p = .007$, $\eta_p^2 = .03$. Independent samples *t*-tests comparing AUC thresholds between experiments at each block found no significant difference at Block 1, $t(168.84) = 0.03$, $p = .972$, $d = 0.00$, 95%CI[-0.28, 0.29], but significant differences at Block 2, $t(173.91) = 2.43$, $p = .016$, $d = 0.35$, 95%CI[0.07, 0.64], and Block 3, $t(164.69) = 3.05$, $p = .003$, $d = 0.45$, 95%CI[0.15, 0.73]. The increase in individual AUC threshold was more pronounced in Experiment 1 than Experiment 2 (see Figures S1A/B).

Within Crowds. An ANOVA with Block (1, 2, 3) and Experiment (1, 2) as factors and the AUC threshold of the within crowds as the dependant variable showed a significant main effect of Block, $F(1.68, 326.36) = 7.00$, $p = .002$, $\eta_p^2 = .035$, with the optimal threshold tending to reduce over the course of each block. The main effect of Experiment was non-significant, $F(1, 194) = 0.73$, $p = .394$, $\eta_p^2 = .004$, as was the interaction between the two factors, $F(1.68, 326.36) = 2.22$, $p = .119$, $\eta_p^2 = .011$.

References

Alenezi, H. M., Bindemann, M., Fysh, M. C., & Johnston, R. A. (2015). Face matching in a long task: Enforced rest and desk-switching cannot maintain identification accuracy. *PeerJ*, *3*, e1184. https://doi.org/10.7717/peerj.1184

MATLAB. (2024). *24.2.0 (R2024b)*. In The MathWorks Inc.

Stanislaw, H., & Todorov, N. (1999). Calculation of signal detection theory measures. *Behavior Research Methods, Instruments, & Computers*, *31*(1), 137-149. https://doi.org/10.3758/bf03207704
